# MitoAMPK inhibits the Warburg effect by MZF1–SIRT6 with glycosis related genes in NSCLC

**DOI:** 10.1111/jcmm.70053

**Published:** 2024-09-03

**Authors:** Shangyu Li, Jinyao He, Lijie Zhang, Qiaojiajie Zhao, Shuqi Zhao, Shanshan Jiang

**Affiliations:** ^1^ Institute of Hematological Research, Shaanxi Provincial People's Hospital Xi'an China; ^2^ Department of Clinical Laboratory Diagnostics Xi'an Medical University Xi'an China

**Keywords:** glycolysis, mitochondrial AMPK, MZF1, non‐small‐cell lung cancer, SIRT6, Warburg effect

## Abstract

MitoAMPK was proved to inhibit the Warburg effect, but the specific mechanisms on non‐small‐cell lung cancer remain unclear. Here, we selected SIRT6 and MZF1 to clarify the mechanism. By western blotting, quantitative polymerase chain reaction, the CCK‐8 assay, and immunohistochemistry assays, we found SIRT6 expression was lower in NSCLC tissues and cell lines than normal tissues and cells. Moreover, SIRT6 could inhibit the Warburg effect by regulating glycolysis‐related genes of SLC2A2, SLC2A4 and PKM2. Finally, we demonstrated the interaction between SIRT6 and MZF1 using ChIP‐qPCR. In conclusion, mitoAMPK inhibits the Warburg effect by regulating the expression of the MZF1–SIRT6 complex.

## 1 INTRODUCTION

The Warburg effect was proved to provide most energy in multiple cancer cells despite the cancer cell having fully functioning mitochondria. Lung cancer is associated with the highest mortality rate worldwide, and 85% of these cases are non‐small‐cell lung cancer (NSCLC).^1^, ^2^ Thus, identifying methods that inhibit the Warburg effect in NSCLC represents a promising treatment approach. In recent years, many studies have focused on the Warburg effect in NSCLC. For example, OTUB2 could bind to and deubiquitinate U2AF1 and then promote NSCLC progression via the AKT/mTOR pathway.^3^ Hyperbaric oxygen therapy and microRNA 101 have also been proven to repress the Warburg effect by HIF‐1α in NSCLC cells.^4^, ^5^ However, the inhibition is still weak, and therefore, a deeper understanding of the mechanisms underlying the Warburg effect is essential to intercept the energy supply and consequently inhibit NSCLC proliferation.

Sirtuin 6 (SIRT6) reportedly suppresses age‐related diseases, such as metabolic diseases and cancer. It can reportedly inhibit the expression of HIF‐1α, LDH, GLUT1, HK, and many other glycolytic genes and consequently inhibit the Warburg effect.^6^ It also reportedly induces autophagy by activating endoplasmic reticulum stress.^7^ However, the tumour suppression effect of SIRT6 in NSCLC was controversial, and little is known about its function in the Warburg effect in NSCLC.

In our previous study, we found that AMPK was located not only in the cytoplasm but also on the mitochondria. Moreover, cytoplasmic AMPK was related to mitochondrial fission and fusion, whereas mitochondrial AMPK (mitoAMPK) participated in the regulation of energy metabolism such that it could promote the aerobic oxidation of A549 and inhibit the Warburg effect.^8^ Using gene array analysis, we found that SIRT6 showed increased expression in A549 cells treated with metformin, which is the activator of AMPK. Considering the proven association of SIRT6 with glycolysis and that MZF1 is an upstream transcription factor of SIRT6, we hypothesized that mitoAMPK can inhibit the Warburg effect via the MZF1–SIRT6 pathway in NSCLC.

To verify our hypothesis, we first compared the expression of mitoAMPK, MZF1, SIRT6, and Warburg effect‐related genes between NSCLC cell lines and normal lung epithelial cells; then, we confirmed the function of MZF1 and SIRT6 in regulating glycolysis and the NSCLC cell phenotype. Finally, we explored the regulatory effect of mitoAMPK in the MZF1–SIRT6 pathway.

## 2 MATERIALS AND METHODS

### 2.1 Materials

All normal lung epithelial cell lines and NSCLC cell lines were obtained from professor Huang's lab. The A549–mito‐AMPK cell line has been previously constructed,^8^ the A549‐MZF1, A549‐SIRT6, HBE‐MZF1, Beas2B‐MZF1, mito‐AMPK‐shRNA‐SIRT6 and mito‐AMPK‐MZF1 were constructed by lenti‐virus infection. The supplier information for experimental materials used in the study is as follows: anti‐SIRT6 and actin antibodies: Proteintech (Wuhan, China); anti‐MZF1: Biorbyt Ltd (Cambridge, UK); anti‐AMPKα: Cell Signalling Technology (MA, USA); secondary antibodies: ZSGB‐BIO (Beijing, China); the LDH assay kit, the ATP determination kit, the HK determination kit and the glucose determination kit: Solarbio Science & Technology (Beijing, China); doxycycline: MedChemExpress (New Jersey, USA); cell lysis buffer for Western blotting and immunoprecipitation: Beyotime (Shanghai, China); the RNeasy Mini Kit: Qiagen (MD, USA); the FastKing RT Kit (with gDNase): Tiangen Biotech (Beijing, China); TB Green Premix Ex Taq II (Tli RNaseH Plus): Takara Biomed (Beijing, China); and mitochondrial protein extraction kits: Sangon Biotech (Shanghai, China), the Lenti‐Easy Packaging Mix: GeneChem (Shanghai, China).

## 3 METHODS

### 3.1 Literature review

Published articles on lung cancer and SIRT6 were searched for in PubMed and EMBASE using the following terms: “sirtuin 6” or “SIRT6” AND “lung cancer” or “lung neoplasm” or “lung tumour” or “lung carcinoma.” The last search was performed on 23 January 2023.

### 3.2 Immunohistochemistry (IHC)

The paraffin sections of NSCLC tissues were roasted at 70°C for 90 min and then sequentially soaked in xylene 1, 2, 3 for 10 min; absolute alcohol, 95% alcohol, and 85% alcohol for 5 min. After washing the sections under flowing water, the endogenous catalase was eliminated by 3%hydrogen peroxide treatment boiling for 15 min. Then, the slides were retrieved with citric acid buffer for 15 min and incubated overnight with SIRT6 primary antibody at 4°C. The next day, the slides were incubated with the second antibody at 37°C for 1 h (PV‐6000, ZSGB‐BIO, Beijing, China) and then incubated with 3,3′‐diaminobenzidine and haematoxylin for 1 min. Finally, the staining intensity and percentage of positive staining were evaluated by semi‐quantitative analysis using a light microscope according to the immunoreactive score.

### 3.3 Stable cell lines construction

293 T cells were seeded into 10 cm dishes. Next day, lenti‐virus were packaged by Lenti‐Easy Packaging Mix and target plasmid using Lipofectamine 2000. The supernatant was collected after 48 h and filtered by 0.22 μm filter. Then we infected target cell lines with polybrene for 8 h, and changed the medium. The adequate concentration of puromycin was added to select the cells which were infected successfully.

### 3.4 Western blotting

Western blotting was performed according to a previously described protocol.^9^ The total protein was separated using 10% sodium dodecyl sulfate gel electrophoresis and transferred to polyvinylidene fluoride membranes (Millipore, MA, USA) at a constant current of 200 mA for 2 h; the membranes were then blocked using 5% non‐fat milk for 1 h. Next, the membranes were incubated overnight with primary antibodies at 4°C. On the next day, after washing with TBST for three times, the membranes were incubated with the secondary antibodies at room temperature for 2 h. Finally, the membranes were exposed, and images were acquired using FluorChem HD2 (Alpha Innotech, San Francisco, CA, USA).

### 3.5 Reverse transcription‐quantitative polymerase chain reaction (RT‐qPCR)

The mRNA was extracted with the RNeasy Mini Kit according to the manufacturer's instructions and then reversed to cDNA using the FastKing RT Kit (with gDNase). Next, qPCR was performed after diluting the cDNA 10 times. The specific primers were as follows: forward 5′‐GTCCGGCAGTCTTCCAGTG‐3′ and reverse 5′‐AAGGTGGTGTCGAACTTGGG‐3′ for SIRT6 (a product of 137 bp); forward 5′‐GCTGCTGCCCTAGTAGATGG‐3′ and reverse 5′‐CCCAGTGGTGATTCCTGCAT‐3′ for MZF1 (a product of 197 bp); forward 5′‐ATGTCGAAGCCCCATAGTGAA‐3′ and reverse 5′‐TGGGTGGTGAATCAATGTCCA‐3′ for PKM (a product of 118 bp); forward 5′‐TCTCCAACTGGACGAGCAAC‐3′ and reverse 5′‐CAGCAGGAGGACCGCAAATA‐3′ for *Homo sapiens* solute carrier family 2 member 4 (SLC2A4, a product of 101 bp); forward 5′‐TCACTGTCGTGTCGCTGTTT‐3′ and reverse 5′‐GATGGCCACGATGCTCAGAT‐3′ for SLC2A1 (a product of 158 bp); and forward 5′‐GAAGGTCGGAGTCAACGGAT‐3′ and reverse 5′‐CTTCCCGTTCTCAGCCATGT‐3′ for GAPDH (a product of 133 bp); forward 5′‐GCTGTCCGGCTCTGTCCTT‐3′ and reverse 5′‐GAGTTCTCCAGTCACCTCTAAA‐3′ for SIRT6 (a product of 100 bp, for ChIP).

### 3.6 CCK‐8 assay

For the CCK‐8 assay, 1500 cells per well were seeded into a 96‐well plate, and OSS_128,167 was added on the next day if necessary, and 10 mL of CCK‐8 was added to each well at the time points of 24, 48 and 72 h. After incubating for 1 h at 37°C in a CO_2_ incubator, the OD value was measured at the wavelength of 450 nm.

### 3.7 Clone formation assay

For the clone formation assay, 200 cells per well were seeded into six‐well plates, and 2 mg/mL doxycycline was added to intervention group wells on the next day. The doxycycline‐containing medium was changed every 3 days, and then, the cells were dyed with Wright–Giemsa solution after the cells were seeded for 15 days. Finally, the images were scanned using a scanner (Colour Laser Jet Pro MFP, M281fdw, hp, China).

### 3.8 LDH assay

Herein, 10^4^ cells were seeded into six‐well plates, and 2 mg/mL doxycycline was added on the next day if necessary. After 24 h, we collected the cells, added 2 mL of the extracting solution, and crushed the cells by ultrasonication. The next steps were performed as per the manufacturer's instructions. Samples, solution 1, and solution 2 were added into experimental tubes. Solution 1, deionized water, and samples were added into control tubes. Different concentrations of the standard solution, solution 1, and deionized water were added into standard tubes. Next, the tubes were incubated at 37°C in a water bath for 15 min, and then, solution 3 was added, and the tubes were incubated again under the same conditions. Finally, we added solution 4 and measured the OD value at 450 nm wavelength.

### 3.9 ATP determination assay

The ATP determination assay was performed following the manufacturer's instructions. In short, 10^6^ cells were harvested and 200 μL extracting solution was added, then sonicated with Sonifier. Next, centrifuged at 10000 × g for 10 min at 4°C, added 500 mL chloroform and centrifuged at 10000 × g for 3 min at 4°C. The supernatant was collected for measurement. Then we prepared a 96‐well plate in which we added 100 μL samples or standard 10 μmol/mL ATP solution, 640 μL Solution 1, 100 μL Solution 2, 100 μL Solution 3, 10 μL Solution 4, 40 μL Solution 5 and 10 μL Solution 6 after mixing and measuring the OD value at 340 nm. After this, we incubated the samples at 37°C for 3 min and measured the OD value once again. The ATP concentration (μmol/10^6^ cell) = 0.125 × △A_measure_ ÷ △A_standard_. △A_measure_ = A_measure_ − A_blank_, △A_standard_ = A_standard_ − A_blank_.

### 3.10 Glucose determination assay

The cell disposal of glucose determination assay is similar with ATP determination assay, cells were broken with distilled water and sonicated, then boiled for 10 min, at last, centrifuged at 8000 × g for 10 min. The supernatant was collected for measurement. Prepared a 96‐well plate, added 20 μL samples or standard 2 μmol/mL glucose solution, 90 μL Solution 2 and 90 μL Solution 3, after mixing, measured the OD value at 505 nm. Then measured the OD value after incubating the samples at 37°C for 15 min. The glucose concentration (μmol/10^6^ cell) = 0.4 × △A_measure_ ÷ △A_standard_. △A_measure_ = A_measure_ − A_blank_, △A_standard_ = A_standard_ − A_blank_.

### 3.11 Hexokinase (HK) determination assay

10^6^ cells were extracted by HK extraction solution, then sonicated and centrifuged to harvest the supernatant for measurement. Prepared the Solution 2 first by Solution 2A: Solution 2B: Solution 2C: Solution 2D = 100 μL: 500 μL: 150 μL: 150 μL. Prepared a 96‐well plate, added 10 μL samples, 180 μL Solution 2 and 10 μL Solution 3, after mixing, measured the OD value at 340 nm (A1). Then measured the OD value after incubating the samples at 37°C for 5 min (A2). The glucose concentration (μmol/10^6^ cell) = 643 × △A ÷ N, △A = A2 − A1.

### 3.12 ChIP‐qPCR

The ChIP‐qPCR assay was performed according to the manufacturer's protocol: first, washed the strip wells with CP1, then added 100 μL CP2, 1 μL of Non‐immune IgG as the negative control or 4 μg MZF1 antibody, covered the wells with Parafilm M and incubated for 90 min at RT. 10^7^ cells were harvested and washed by PBS, then fixed the cells with 1% formaldehvde for 10 min at RT. 1 mL of 1.25 M Glycine solution were added and then centrifuged, washed by ice‐cold PBS. 500 μL CP3A was added and incubated with ice for 10 min, next, added CP3B containing Protease Inhibitor Cocktail to re‐suspend the nuclear pellet, after incubated with ice, sheared DNA by sonication. After incubated DNA supernatant and strip wells with MZF1 antibody for 90 min at RT, added CP5‐CP7 to reverse the cross‐linked DNA and purify the DNA. Subsequently, eluted the purified DNA with 20 μL CP8. Finally, the qPCR was performed with the SIRT6 primer mentioned above.

### 3.13 Statistical analysis

Data were expressed as mean ± SEM. and the difference was analysed by Student's *t*‐test and two‐way ANOVA using the GraphPad Prism software. A *p* < .05 was considered to indicate significant difference.

## 4 RESULTS

### 4.1 The association between SIRT6 expression and survival of patients with NSCLC

**FIGURE 1:**
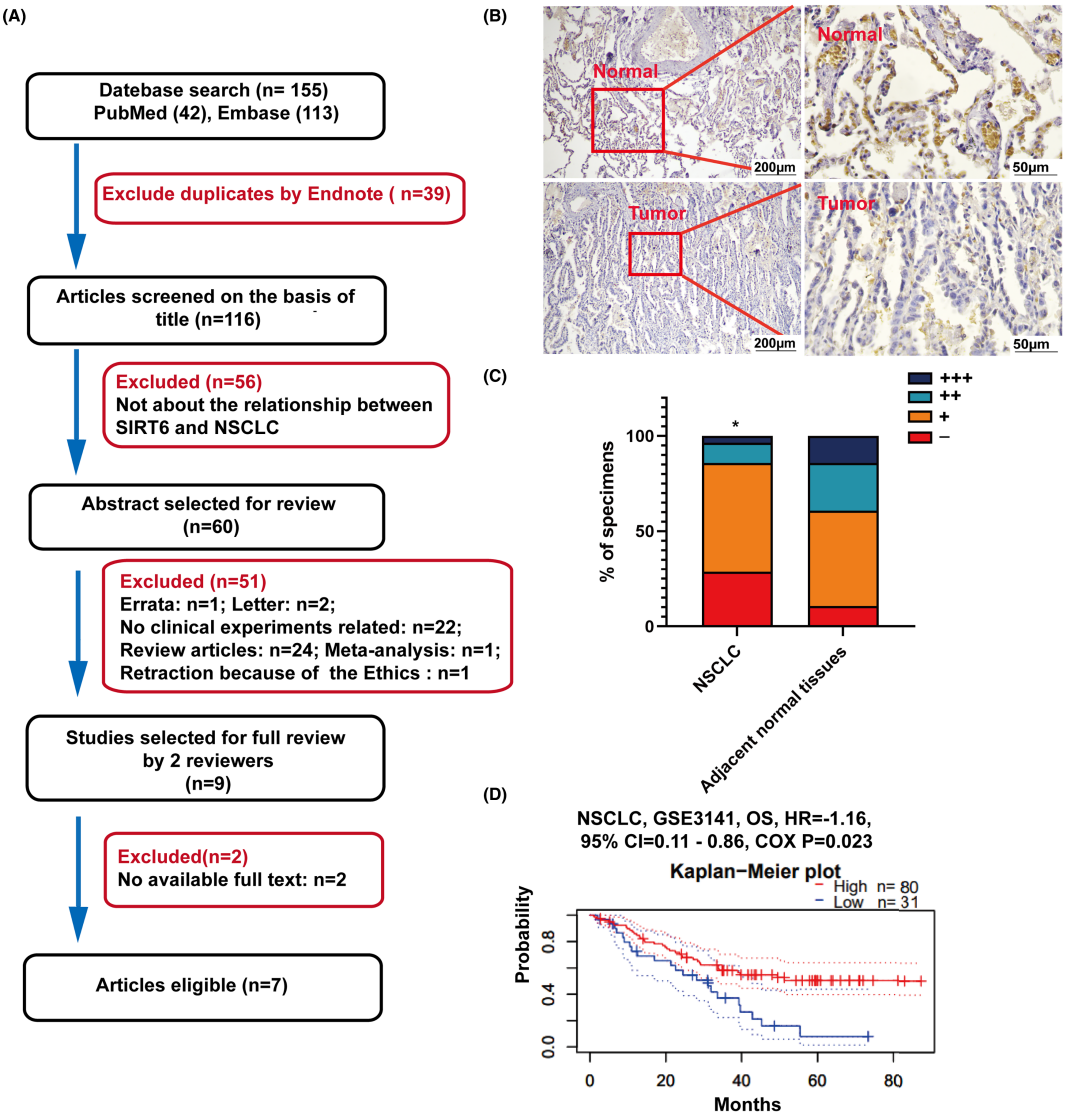
SIRT6 expression and the prognosis in NSCLC and adjacent normal tissues: (A) flow chart of literature collection, screening, exclusion, and inclusion. (B) The tissue specimens showed were from one patient with SIRT6 expression by IHC (magnification, left: ×100 and right: ×400). (C) The percentage of SIRT6 expression in 38 NSCLC tissues in comparison with adjacent normal tissues. −, the staining index score <2; +, 3< the score <5; ++, 6< the score <8; +++, 9< the score <12. (D), Kaplan–Meier survival curves from the PrognoScan database. *p < 0.05.

As there have been conflicting reports on the expression of SIRT6 in NSCLC tissues, we analysed their relationship by performing a meta‐analysis. We selected 155 studies on the association between SIRT6 expression and NSCLC. After excluding duplicate publications, letters, review articles, studies that did not use patient tissues, and articles with full text not available, seven studies were chosen (Figure 1A, Table 1). However, we analysed the seven related studies obtained after applying all criteria. Among these, three studies reported higher SIRT6 expression in NSCLC tissues than in tumour‐adjacent tissues, whereas the other four studies reported opposite results. To verify their relationship between SIRT6 expression and NSCLC by ourselves, thirty‐eight patients diagnosed as NSCLC at Shaanxi Provincial People's Hospital from January 2019 to December 2021 were included, and their NSCLC and adjacent normal tissues were subjected to IHC assay of SIRT6. The results revealed higher SIRT6 expression in adjacent normal tissues than in NSCLC tissues (Figure 1B,C). Moreover, high SIRT6 expression was also associated with better prognosis according to an analysis using the PrognoScan database (http://dna00.bio.kyutech.ac.jp/PrognoScan/, Figure 1D).

**TABLE 1:** The characteristics of the eligible studies about SIRT6 expression and NSCLC in meta‐analysis.

Author	Year	Region	Method	Title	SIRT6 expression in NSCLC than normal tissues	Number of samples	Reference
Azuma	2015	Japan	IHC	SIRT6 expression is associated with poor prognosis and chemosensitivity in patients with non‐small cell lung cancer	Higher expression	98	[10]
Chen	2017	China	IHC	RASSF1A and SIRT6 in non‐small cell lung cancer: Relationship with clinical outcome	Lower expression	122	[11]
Han	2014	China	Western blotting and qPCR	Sirtuin SIRT6 suppresses cell proliferation through inhibition of Twist1 expression in non‐small cell lung cancer	Lower expression	36	[12]
Xiong	2021	China	Western blotting and qPCR	SIRT6 through PI3K/Akt/mTOR signalling pathway to enhance radiosensitivity of non‐small cell lung cancer and inhibit tumour progression	Lower expression	50	[13]
Ruan	2018	China	Western blotting and qPCR	miR‐34a inhibits tumorigenesis of NSCLC via targeting SIRT6	Higher expression	4	[14]
Zhu	2018	China	Western blotting and qPCR	Downregulation of SIRT6 is associated with poor prognosis in patients with non‐small cell lung cancer	Lower expression	86	[15]
Bai	2016	China	IHC and Western blotting	Upregulation of SIRT6 predicts poor prognosis and promotes metastasis of non‐small cell lung cancer via the ERK1/2/MMP9 pathway	Higher expression	12	[16]

**FIGURE 2:**
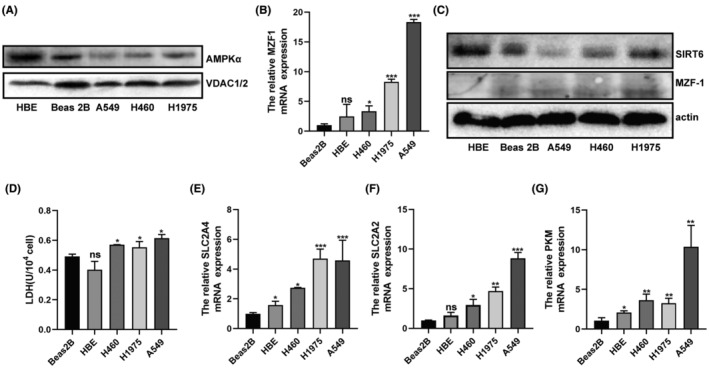
Mitochondrial AMPK (mitoAMPK), SIRT6, and MZF1 showed different expressions in NSCLC cell lines when compared with normal lung epithelial cells, and the NSCLC cell lines showed a much stronger Warburg effect. (A) The mitochondrial protein of Beas2B, HBE, H460, H1975, and A549 cells were extracted, and the mitoAMPK protein expression was detected by Western Blotting. (B) The relative MZF1 mRNA expression of Beas2B, HBE, H460, H1975, and A549 cells was measured by q‐PCR. (C) Western blotting was performed to detect SIRT6 and MZF1 protein expression. (D) The LDH concentration of Beas2B, HBE, H460, H1975, and A549 cells was displayed at the wavelength of 450 nm. (E–G) The relative SLC2A4, SLC2A2, and PKM mRNA expressions of Beas2B, HBE, H460, H1975, and A549 cells were measured using q‐PCR. *p < 0.05, **p < 0.01, ***p < 0.001.

MitoAMPK, SIRT6, and MZF1 were differently expressed in NSCLC in vitro, and the NSCLC cell lines showed stronger Warburg effect. The mitochondrial proteins of lung normal epithelial cell lines (HBE and Beas2B) and NSCLC cell lines (H460, H1975 and A549) were extracted, and Western blotting showed that mitoAMPK had higher expression in normal lung epithelial cell lines (Figure 2A). By detecting the protein and mRNA expressions of SIRT6 and MZF1 in the five cell lines, we found that SIRT6 showed higher expression and MZF1 showed lower expression in the normal lung epithelial cell lines HBE and Beas2B than in NSCLC cell lines (Figure 2B,C). Moreover, compared with normal lung epithelial cell lines, NSCLC cell lines produced more LDH (Figure 2D) and expressed more glycolysis‐related genes, such as SLC2A4, SLC2A2 and PKM (Figure 2E,G).

### 4.2 MZF1 and SIRT6 showed opposite effect on NSCLC cell phenotype

**FIGURE 3:**
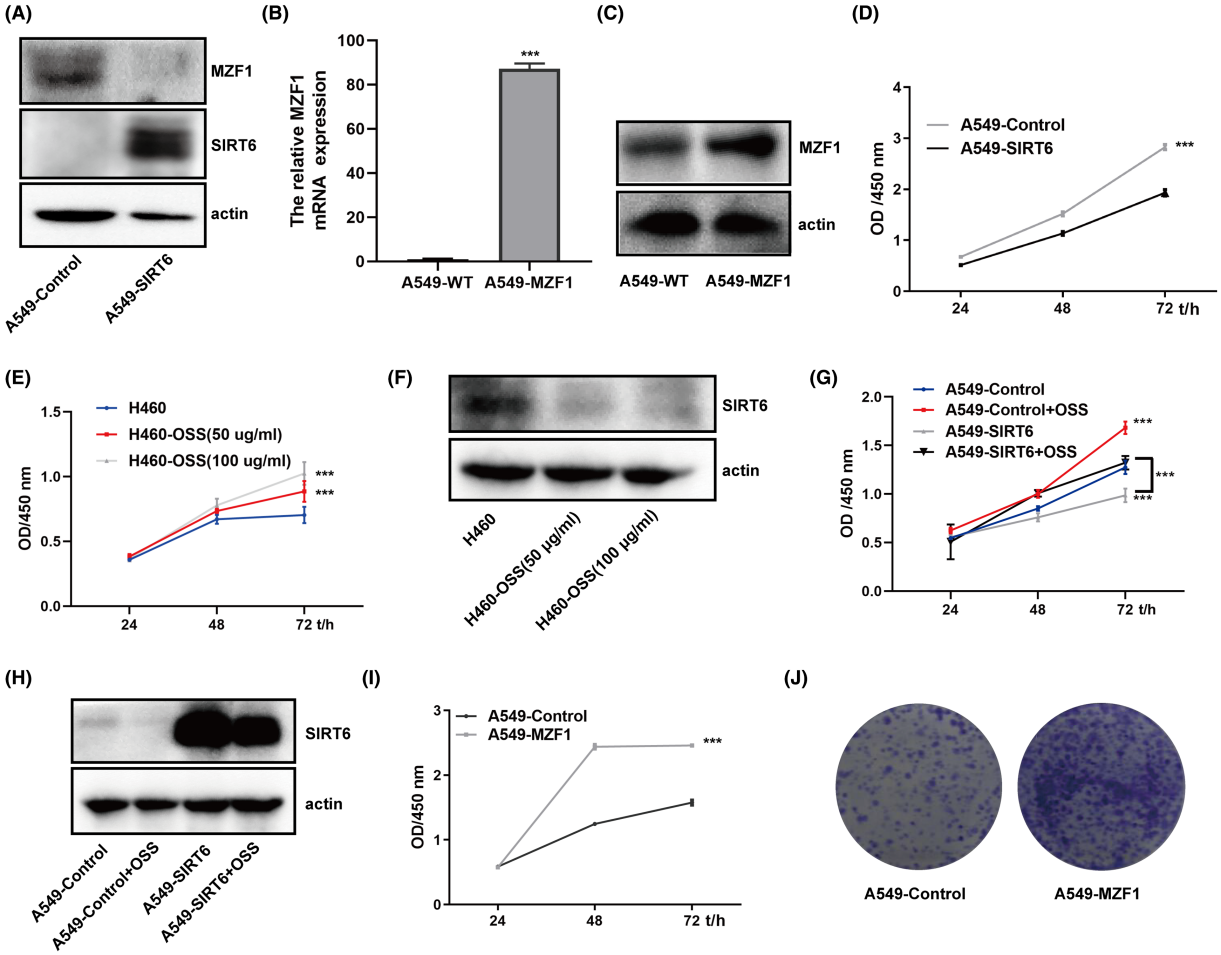
MZF1 and SIRT6 showed the opposite effect on the NSCLC cell phenotype. Stable MZF1 and SIRT6 cell lines were constructed using a lentivirus: (A) The protein expression of SIRT6 and MZF1 in A549‐SIRT6 was detected by Western blotting. (B, C) The relative MZF1 mRNA and MZF1 protein expressions in A549‐MZF1 were measured by q‐PCR and Western blotting. (D, F) 1500 cells/well were seeded into 96‐well plates, and the OD values were detected at a wavelength of 450 nm at 24, 48 and 72 h. (E) Similarly, 1500 cells/well were seeded into 96‐well plates, and on the next day, the cells were treated using 50 μg/mL and 100 μg/mL OSS_128,167, and then, the OD values were detected at a wavelength of 450 nm at 24, 48 and 72 h. (F) The protein expression of SIRT6 was detected by Western blotting after treating H460 cells with 50 μg/mL and 100 μg/mL OSS_128,167 for 48 h. (G) 1500 A549‐Control and A549‐SIRT6 cells/well were seeded into 96‐well plates, and on the next day, the cells were treated 100 μg/mL OSS_128,167, and then, the OD values were detected at a wavelength of 450 nm at 24, 48 and 72 h. (H) The protein expression of SIRT6 was detected by Western blotting after treating H460 cells with 100 μg/mL OSS_128,167 for 48 h. (I) 1500 cells/well of A549‐WT and A549‐MZF1 were seeded into 96‐well plates, and the OD values were detected at a wavelength of 450 nm at 24, 48,and 72 h. (J) 200 cells were seeded into six‐well plates, and the formed clones were stained with Wright‐Giemsa solution and photoimaged. ***p < 0.001.

Considering that MZF1 and SIRT6 were differently expressed in NSCLC cell lines and normal lung epithelial cell lines and that the function of SIRT6 in cancers remains controversial, we first tried to elucidate the function of MZF1 and SIRT6 in A549 and H460 cells. We constructed an MZF1‐overexpressing A549 cell line (A549‐MZF1) and a SIRT6‐overexpressing A549 cell line (A549‐SIRT6). Figure 3A,C show that the cell lines were successfully constructed. Notably, A549‐SIRT6 cells showed slower proliferation (Figure 3D); However, upon treatment with the SIRT6 inhibitor OSS_128,167, the SIRT6 protein expression was inhibited and the proliferation of H460 increased dose‐dependently, same results were showed in A549‐SIRT6 cells (Figure 3E,H); Conversely, A549‐MZF1 cells shower higher proliferation than A549 cells (Figure 3I), and this finding was upheld by the clone formation assay results (Figure 3J).

**FIGURE 4:**
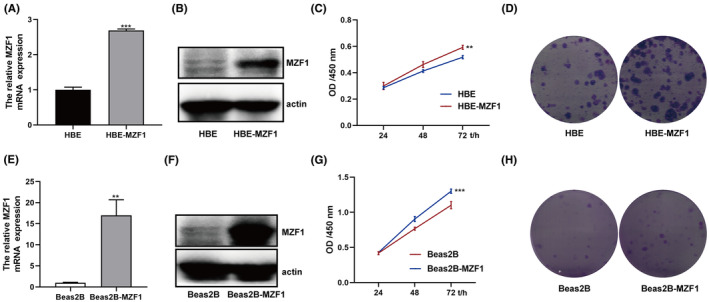
The function of MZF1 in normal lung epithelial cells. Stable MZF1 cell lines HBE‐MZF1 and Beas 2B‐MZF1 cells were constructed using a lentivirus: (A, B, E, F) The relative MZF1 mRNA and protein expression were tested by qPCR and Western blotting. (C, G) 1500 cells/well were seeded into 96‐well plates, and the OD values were detected at a wavelength of 450 nm at 24, 48 and 72 h. (D, H) 200 cells were seeded into six‐well plates, and the formed clones were stained with Wright‐Giemsa solution and photoimaged. **p < 0.01, ***p < 0.001.

Moreover, as MZF1 was high expressed in NSCLC cells, so only over‐expressed its expression is inadequate to explain the function of MZF1 in promoting NSCLC cell growth, so we over‐expressed MZF1 expression in HBE and Beas2B cells, Figure 4A,B,E,F showed that the cell lines were constructed successfully, the CCK‐8 assays and clone formation assays demonstrated that higher MZF1 expression could faster normal lung epithelial cells growth, which provided the evidence that MZF1 could promote NSCLC cell proliferation (Figure 4C,D,G,H).

### 4.3 The relationship of MZF1 and SIRT6 with glycolysis

**FIGURE 5:**
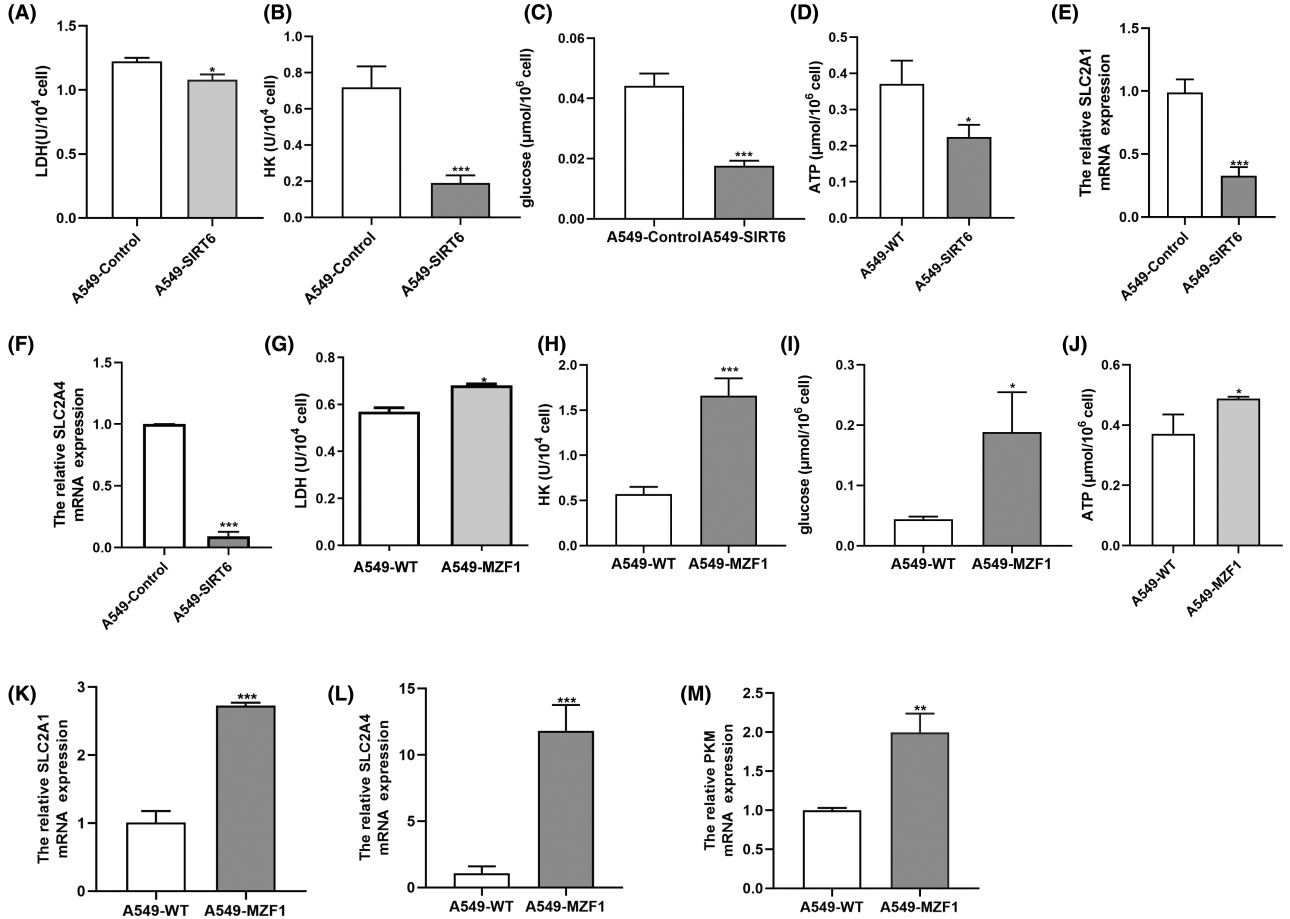
The relationship among MZF1, SIRT6, and glycolysis in NSCLC. (A–D, G–J) The concentration of LDH, HK, glucose and ATP in A549‐SIRT6 and A549‐MZF1 was detected following the manufacturer's instructions. (E, F, K–M): The relative expressions of SLC2A4, SLC2A2 and MZF1 mRNA were detected by q‐PCR. *p < 0.05, **p < 0.01, ***p < 0.001.

**FIGURE 6:**
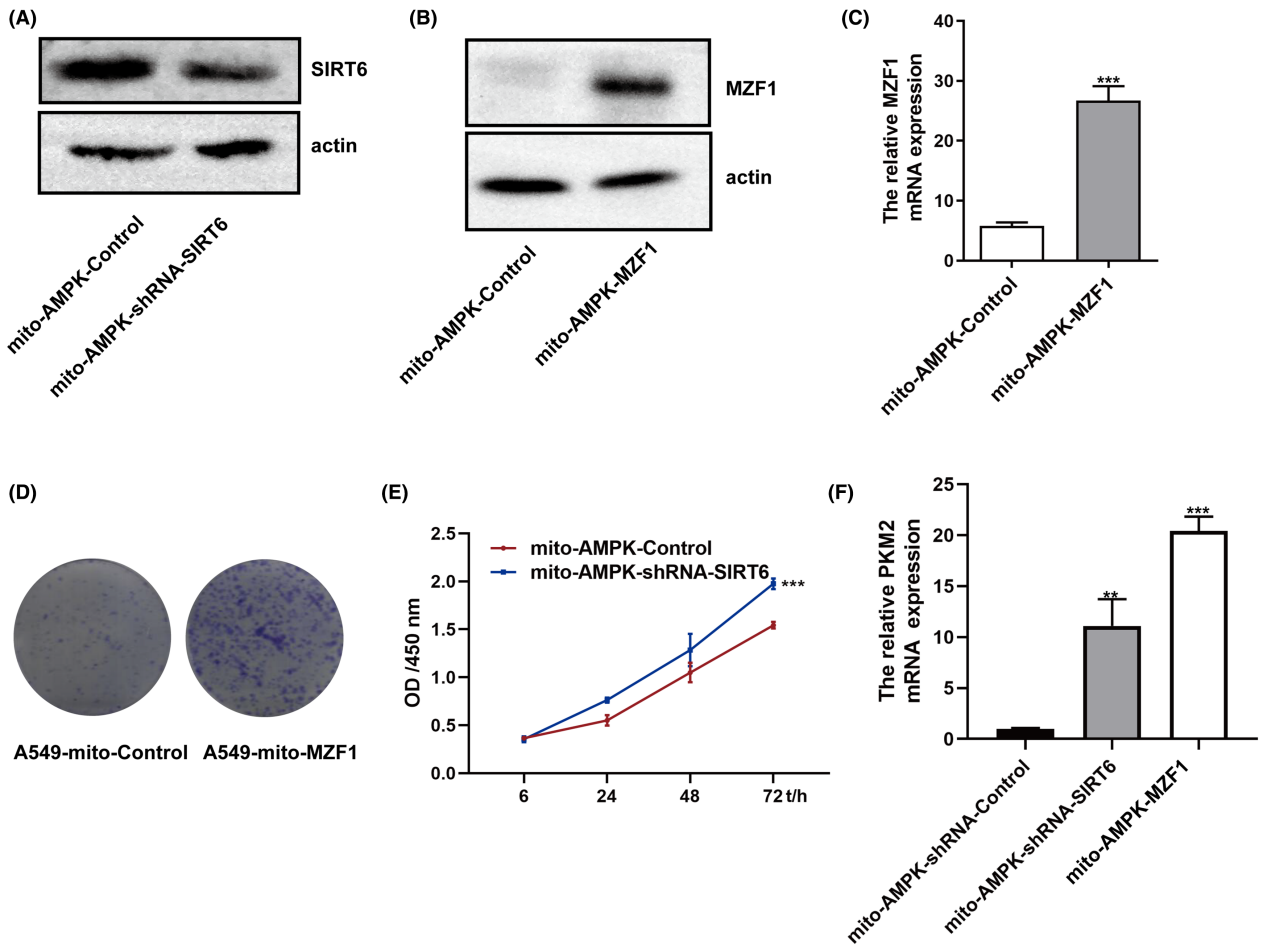
The function of MZF1, SIRT6, and glycolysis in mitochondrial AMPK cells. (A–C) The mito‐AMPK‐shRNA‐SIRT6 and mito‐AMPK‐MZF1 cell lines were verified by Western blotting and q‐PCR. (D) The clone formation assay was performed using Wright and Giemsa solution. (E) The CCK‐8 assay was performed as described before. (F) The relative PKM2 mRNA expression was detected by q‐PCR. **p < 0.01, ***p < 0.001.

Notably, no concrete findings have been reported on the correlation between SIRT6 and the Warburg effect, and only two studies have been reported on the relationship between MZF1 and glycolysis; Thus, we explored the correlation between SIRT6 and the Warburg effect. Compared with the A549‐Control cells, A549‐SIRT6 cells showed less productions of LDH, ATP and HK (Figure 5A,B,D), less consumption of glucose (Figure 5C), less expression of SLC2A1 and SLC2A4 (Figure 5E,F). At the same time, we tested these targets in A549‐MZF1 cells. As expected, these cells showed higher expression of all Warburg effect‐related targets, namely LDH, ATP and HK productions, glucose consumption, SLC2A1, SLC2A4, and PKM2 (Figure 5G–M). Next, we constructed a MZF1‐overexpressing cell line (mito‐AMPK‐MZF1) and SIRT6‐underexpressing cell line (mito‐AMPK‐shRNA‐SIRT6) using the A549‐mito‐AMPK cell line (Figure 6A–C). Similar results were found in the mito‐AMPK‐overexpressing cell line (Figure 6D–F). The clone formation assay showed that mito‐AMPK‐MZF1 cells grew faster than normal cells (Figure 6D), and the CCK‐8 assay verified that mito‐AMPK‐shRNA‐SIRT6 had faster proliferation (Figure 6E). Moreover, both mito‐AMPK‐MZF1 and mito‐AMPK‐shRNA‐SIRT6 cell lines had higher PKM2 expression, suggesting that MZF1 increases the Warburg effect and SIRT6 has the opposite effect (Figure 6F).

### 4.4 MitoAMPK inhibits A549 proliferation by glycolysis

**FIGURE 7:**
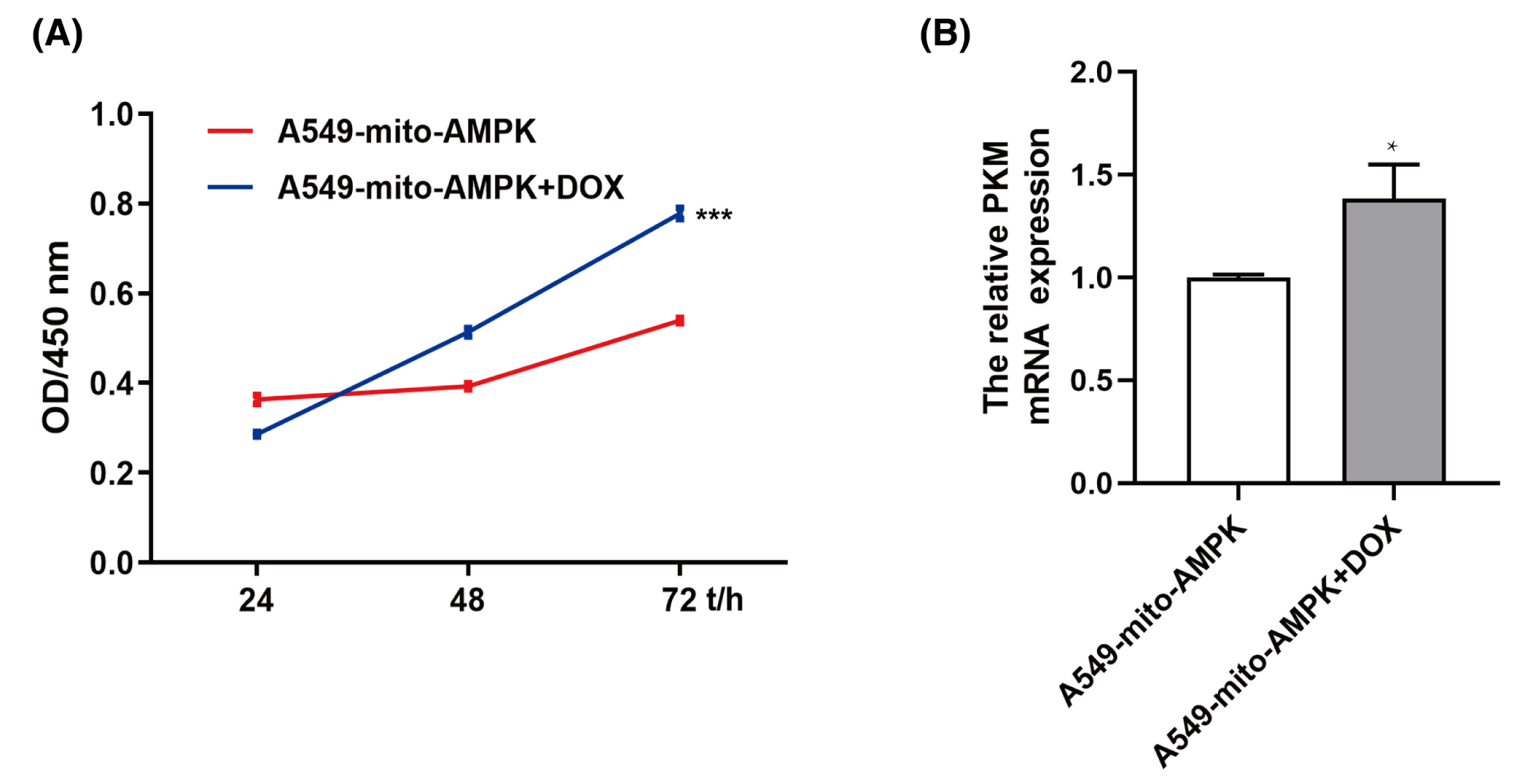
mitoAMPK inhibited A549 proliferation by glycolysis and not by mitochondrial biogenesis or fission. (A) 1500 A549‐mito‐AMPK cells/well were seeded into 96‐well plates, and on the next day, the cells were treated using 2 mg/mL doxycycline, which was followed by the acquisition of OD values at a wavelength of 450 nm at 24, 48 and 72 h. (B) The relative PKM mRNA expression was tested when A549‐mito‐AMPK was treated with 2 mg/mL doxycycline for 24 h. *p < 0.05, ***p < 0.001.

To investigate the relationship between AMPK on mitochondria and tumour, we constructed mitochondria‐specific expression. The AMPK cell A549‐mito‐AMPK is regulated by DOX. That is, when DOX is not added, cells express transfected mitochondrial AMPK (mitoAMPK), and when DOX is added, this specific expression is lost (Jiang et al.).8 As mentioned earlier, mitoAMPK could inhibit A549 proliferation (Figure 7A). Moreover, mitoAMPK could reduce the relative PKM mRNA expression, which confirms that mitoAMPK functions in glycolysis inhibition (Figure 7B).

### 4.5 The function of MZF1–SIRT6 in the Warburg effect was regulated by mitoAMPK in A549 cells

**FIGURE 8:**
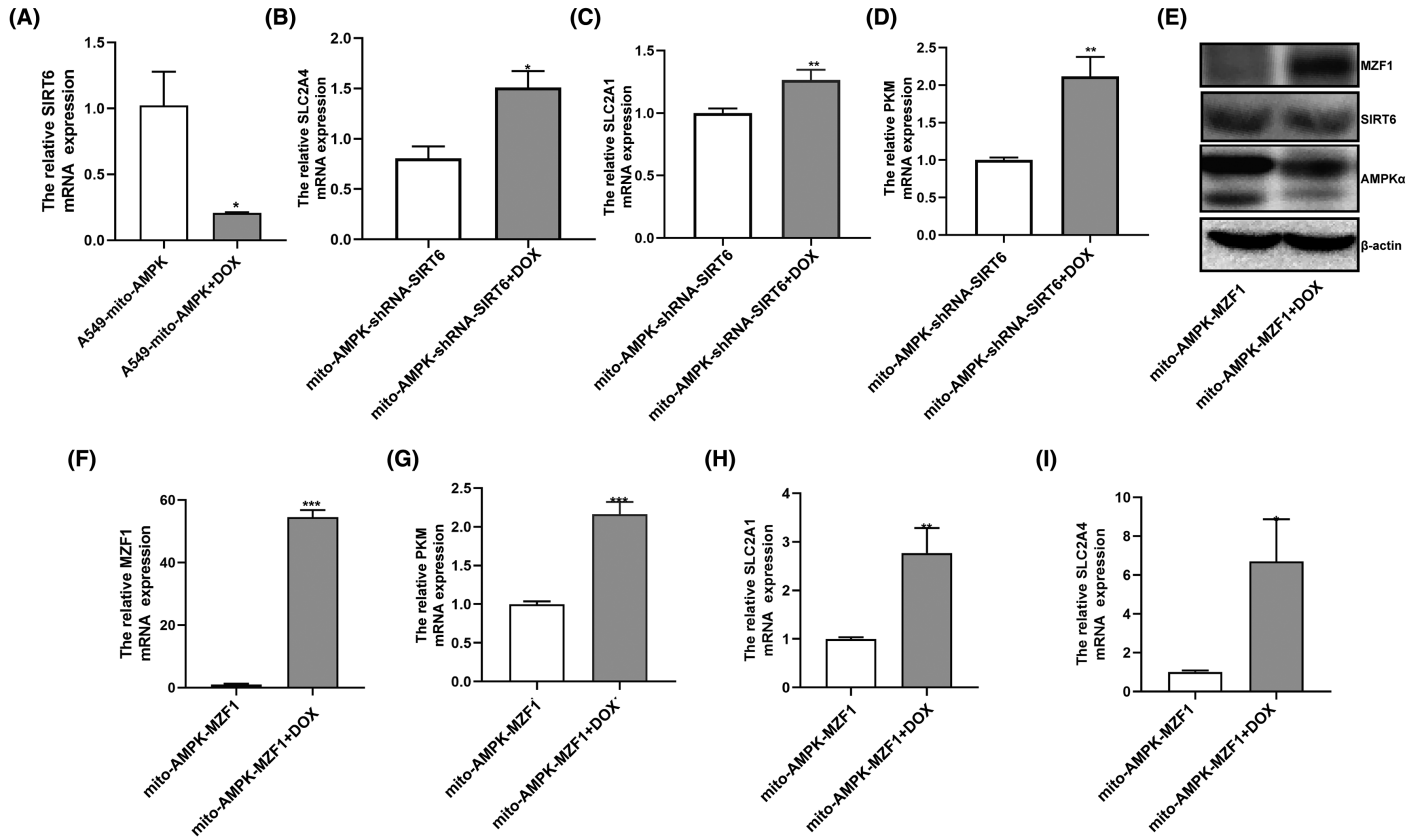
The function of MZF1–SIRT6 on the Warburg effect was regulated by mitoAMPK in A549 cells. (A) The relative SIRT6 mRNA expression of A549‐mito‐AMPK was measured using q‐PCR after the cells were treated with 2 mg/mL doxycycline for 24 h. (B–D) The relative expression of SLC2A4, SLC2A1, and PKM mRNAs was tested when mito‐AMPK‐shRNA‐SIRT6 cells were treated with 2 mg/mL doxycycline for 24 h. (E) The MZF1 and SIRT6 protein expression was detected when mito‐AMPK‐MZF1 cells were treated with 2 mg/mL doxycycline for 24 h. (F–I) The relative expression of MZF1, SLC2A4, SLC2A1 and PKM mRNA were tested when mito‐AMPK‐MZF1 cells were treated with 2 mg/mL doxycycline for 24 h. *p < 0.05, **p < 0.01, ***p < 0.001.

To check if mitoAMPK inhibits the Warburg effect by regulating MZF1–SIRT6, we first treated A549‐mito‐AMPK cells with 2 mg/mL doxycycline to Tet‐Off mitoAMPK expression and found that SIRT6 expression would decrease (Figure 8A); This would mean that SIRT6 was upregulated by mitoAMPK. Then, we treated mito‐AMPK‐shRNA‐SIRT6 cells with 2‐mg/mL doxycycline to Tet‐Off the expression of mitoAMPK,^8^ and glycolysis‐related genes were up‐expressed (Figure 8B–D). Similarly, when we tet‐off mitoAMPK expression in mito‐AMPK‐MZF1 cells, the expression of SIRT6 protein decreased and that of MZF1 mRNA and protein increased (Figure 8E); Furthermore, glycolysis‐related genes were up‐expressed (Figure 8F–I).

### 4.6 Preliminary exploration of the correlation between MZF1 and SIRT6

**FIGURE 9:**
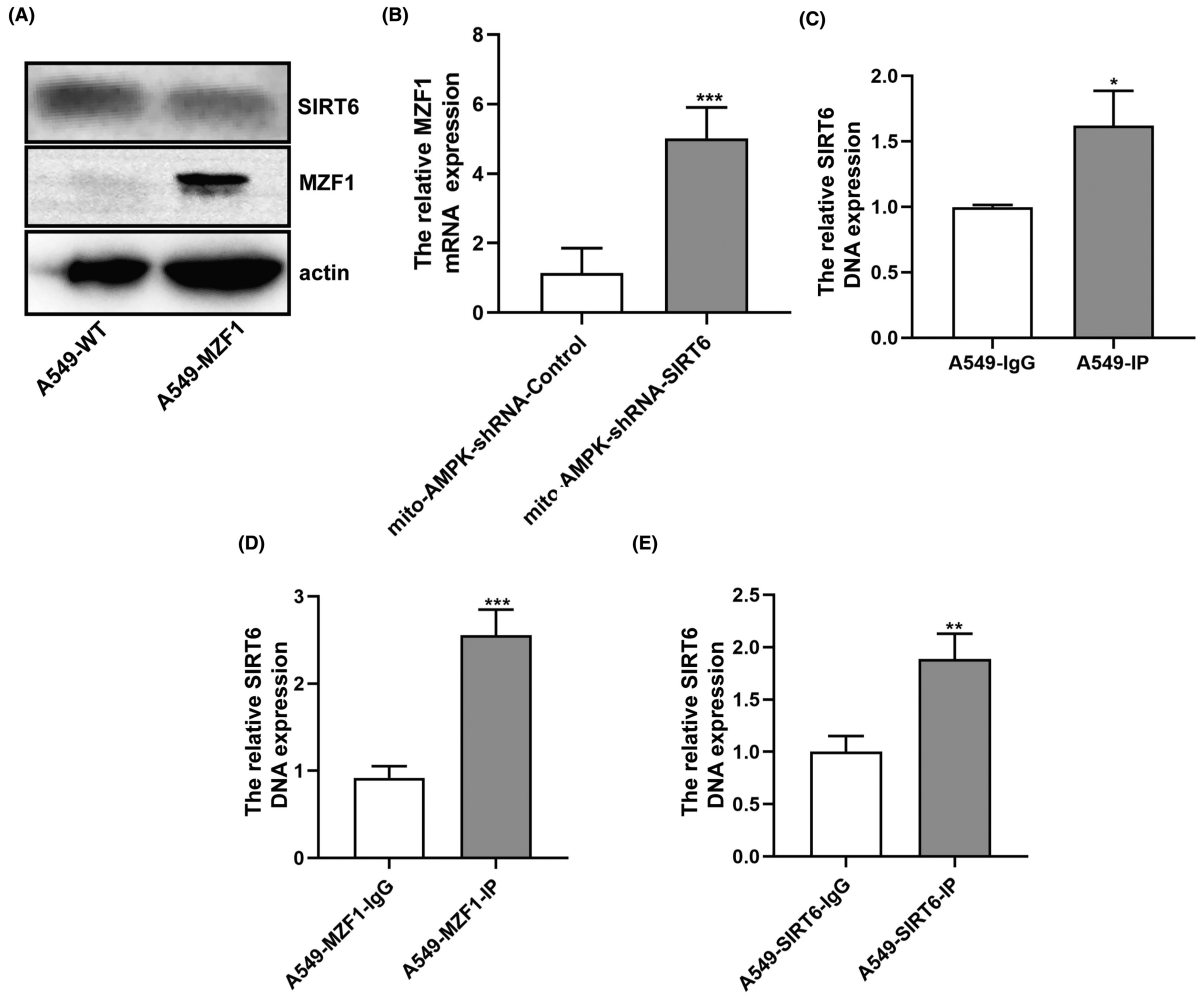
The correlation between MZF1 and SIRT6 was preliminarily explored. (A) The SIRT6 and MZF1 protein expression was detected by Western blotting in A549‐WT and A549‐MZF1 cells. (B) The relative expression of MZF1 mRNA was tested in mito‐AMPK‐shRNA‐SIRT6 and control cells. (C–E) ChIP was performed to test the correlation between MZF1 and SIRT6. *p < 0.05, **p < 0.01, ***p < 0.001.

To test our prediction of MZF1 being the upstream transcription factor of SIRT6, we detected the expression of SIRT6 in A549‐MZF1 cells and found that when the MZF1 protein expression increased, SIRT6 expression decreased (Figure 9A); similarly, when we knocked down SIRT6 expression, MZF1 expression increased (Figure 9B). To verify that MZF1 could combine with the promoter region of SIRT6, we predicted the binding sites at Jaspar (http://jaspar.binf.ku.dk/). It showed that there are 19 binding sites which relative scores are over 0.8. Moreover, ALGGEN (https://alggen.lsi.upc.es/cgi‐bin/promo_v3/promo/promoinit.cgi?dirDB=TF_8.3) was also used to predicted the binding sites, there are 8 binding sites and we chose the one which dissimilarity is 0.00% and verified by ChIP‐qPCR assay. It showed that the binding sequence of MZF1 to SIRT6 is GTGGGGA (the binding site is from 1703 to 1709) at A549, A549‐MZF1 and A549‐SIRT6 cells (Figure 9C–E).

## 5 DISCUSSION

Although most energy needed by cells comes from aerobic oxidation, in cancer cells, glucose gets fermented to lactate and leads to quick release of ATP, which called the Warburg effect. The Warburg effect is the primary way of energy production in several cancer cell lines. There is consensus on the notion that cancer cells cannot grow well if the Warburg effect is inhibited. In NSCLC, numerous factors have been proven to be related to the Warburg effect: OTUB2 could bind to and deubiquitinate U2AF1 and consequently promote NSCLC progression,^3^ and α‐Hederin could inhibit glycolysis in A549 and H460 cells by activating SIRT6.^17^ Our previous study showed that AMPK could be located on the mitochondria, and this mitoAMPK could inhibit the Warburg effect. The gene array displayed that AMPK could increase the SIRT6 mRNA expression. Considering that MZF1 is the upstream transcription factor of SIRT6 and little is known about the relationship among MZF1, SIRT6, and NSCLC, we first investigated the expression of MZF1, SIRT6, and mitoAMPK proteins in NSCLC cell lines.

SIRT6 belongs to the nicotinamide adenine dinucleotide (NAD^+^)‐dependent class III histone deacetylase family and has been proven to influence NSCLC progression and prognosis.^10^ SIRT6 can reportedly promote NSCLC progression and migration: SIRT6 could drive epithelial‐to‐mesenchymal transition by transrepression the expression of KLF4,^18^ and SIRT6 knockdown could improve paclitaxel sensitivity.^10^ However, some studies have also reported that SIRT6 could suppress NSCLC proliferation. It has been proven that MDL‐800, its allosteric activator, inhibits NSCLC cell line proliferation by suppressing the mitogen‐activated protein kinase pathway.^19^ Compared with normal lung tissues, NSCLC tissues showed lower SIRT6 expression, while higher SIRT6 expression was associated with a better prognosis.^11^ As the function of SIRT6 in tumour suppression was controversial, we first tried to prove the relationship between SIRT6 and NSCLC using a meta‐analysis. However, as the specific numbers could not be obtained, we just compared the seven finally included studies and found that three studies reported higher SIRT6 expression in NSCLC and four studies reported lower expression. As no consensus could be reached with these contradictory reports, we verified SIRT6 expression in NSCLC tissues using IHC, and the results showed that SIRT6 expression were higher in adjacent normal tissues than in NSCLC tissues (Figure 1B,C). The cell lines assay returned the same result: SIRT6 protein showed lower expression in NSCLC cell lines than in the normal lung epithelial cells (Figure 2C). When SIRT6 was overexpressed in A549 cells, the cells showed slower proliferation (Figure 3D), and when H460 cells were treated with the SIRT6 inhibitor, the cells grew much more quickly (Figure 3E,G). In the mito‐AMPK‐overexpressing cell line, we knocked down SIRT6, and the cells showed higher proliferation (Figure 6E).

AMPK is a recognized tumour suppressor and shows lower expression in multiple cancers.^20^ It functions as a tumour suppressor by inhibiting HIF‐1α^21^ and promoting cell autophagy (mTOR signalling pathway)^22^ and apoptosis.^23^ Moreover, in our previous study, we found that AMPK could be located in the cytoplasm and mitochondria and that it performed different functions depending on its location. We found no study on the expression of mitoAMPK in NSCLC, and thus, we tested mitoAMPK expression in NSCLC cell lines and found that it showed lower expression in these cells (Figure 2A). The CCK‐8 assay showed that mitoAMPK also had tumour suppressor function; the cells grew faster as we Tet‐Off the mitoAMPK by using doxycycline (Figure 7A). Similarly, we have also studied the expression of MZF1, and the results are in agreement with those of studies wherein increased expression of MZF1 was found in NSCLC cell lines (Figure 2B,C). In addition, it could promote A549 proliferation (Figure 3I,J).

As we previously demonstrated that mitoAMPK inhibited A549 cell proliferation by inhibiting glycolysis, after elucidating the function of MZF1, SIRT6, and mitoAMPK in NSCLC, we tested their relationship with the Warburg effect. First, we wanted to investigate if glycolysis was more likely to occur in NSCLC cell lines than in normal lung cell lines; Therefore, LDH and glycolysis‐related genes were tested, and they indeed showed higher expression in NSCLC cell lines (Figure 2D–G). Next, we verified that SIRT6 could inhibit the Warburg effect, whereas MZF1 had the opposite function (Figure 5). Finally, we showed that the effect of SIRT6‐MZF1 on glycolysis was regulated by mitoAMPK (Figure 8): when we Tet‐Off mitoAMPK expression, SIRT6 expression decreased and MZF1 expression increased, and at the same time, glycolysis increased. Therefore, mitoAMPK was crucial in regulating SIRT6‐MZF1‐induced glycolysis. Although we found that mitoAMPK could inhibit extracellular acidification rate in A549 cells,^8^ the underlying mechanism remains to be elucidated. Herein, we displayed that mitoAMPK could inhibit several glycolysis‐related genes, such as PKM2, SLC2A1, and SLC2A4, and that it could inhibit the production of LDH in A549 cells. Although the presence of an association of SIRT6 and MZF1 with the Warburg effect has been established,^7^, ^24^ this study is the first to demonstrate that they were regulated by mitoAMPK. Taking together, upregulating mitoAMPK could inhibit MZF1, activate SIRT6, and consequently inhibit the Warburg effect. Finally, we have used ChIP‐qPCR to demonstrate that MZF1 could bind with the promoter sequence of SIRT6 at GTGGGGA.

## 6 LIMITATIONS

We verified that mitoAMPK could inhibit the Warburg effect by SIRT6‐MZF1 in A549 cells. However, we did not detect mitoAMPK expression in NSCLC patients' tissues and could not find a way to up‐regulate its expression.

## AUTHOR CONTRIBUTIONS

**Shangyu Li:** Data curation (lead); methodology (lead). **Jinyao He:** Data curation (lead). **Lijie Zhang:** Formal analysis (lead); funding acquisition (supporting). **Qiaojiajie Zhao:** Formal analysis (equal); funding acquisition (supporting); software (lead). **Shuqi Zhao:** Conceptualization (equal); funding acquisition (supporting); supervision (lead); writing – original draft (equal). **Shanshan Jiang:** Conceptualization (lead); funding acquisition (lead); writing – original draft (lead).

## FUNDING INFORMATION

This study was supported by grants from the Natural Science Foundation of Shaanxi Province (2022JQ‐896, 2022JQ‐944, 2021JQ‐909), and the Foundation of Shaanxi Provincial People's Hospital (2021YJY‐25, 2022JY‐55, 2022JY‐56, 2022BJ‐03).

## CONFLICT OF INTEREST STATEMENT

The authors declare to have no conflicts of interest.

## Data Availability

The data that support the findings of this study are available from the corresponding author upon reasonable request.
